# Gaussian-Distributed Spread-Spectrum for Covert Communications

**DOI:** 10.3390/s23084081

**Published:** 2023-04-18

**Authors:** Ismail Shakeel, Jack Hilliard, Weimin Zhang, Mark Rice

**Affiliations:** Information Sciences Division, Defence Science and Technology Group, Edinburgh, SA 5111, Australia

**Keywords:** covert communications, spread-spectrum schemes, communications signal processing, low probability of detection, secure communications, signal constellations

## Abstract

Covert communication techniques play a crucial role in military and commercial applications to maintain the privacy and security of wireless transmissions from prying eyes. These techniques ensure that adversaries cannot detect or exploit the existence of such transmissions. Covert communications, also known as low probability of detection (LPD) communication, are instrumental in preventing attacks such as eavesdropping, jamming, or interference that could compromise the confidentiality, integrity, and availability of wireless communication. Direct-sequence spread-spectrum (DSSS) is a widely used covert communication scheme that expands the bandwidth to mitigate interference and hostile detection effects, reducing the signal power spectral density (PSD) to a low level. However, DSSS signals possess cyclostationary random properties that an adversary can exploit using cyclic spectral analysis to extract useful features from the transmitted signal. These features can then be used to detect and analyse the signal, making it more susceptible to electronic attacks such as jamming. To overcome this problem, a method to randomise the transmitted signal and reduce its cyclic features is proposed in this paper. This method produces a signal with a probability density function (PDF) similar to thermal noise, which masks the signal constellation to appear as thermal white noise to unintended receivers. This proposed scheme, called Gaussian distributed spread-spectrum (GDSS), is designed such that the receiver does not need to know any information about the thermal white noise used to mask the transmit signal to recover the message. The paper presents the details of the proposed scheme and investigates its performance in comparison to the standard DSSS system. This study used three detectors, namely, a high-order moments based detector, a modulation stripping detector, and a spectral correlation detector, to evaluate the detectability of the proposed scheme. The detectors were applied to noisy signals, and the results revealed that the moment-based detector failed to detect the GDSS signal with a spreading factor, *N* = 256 at all signal-to-noise ratios (SNRs), whereas it could detect the DSSS signals up to an SNR of −12 dB. The results obtained using the modulation stripping detector showed no significant phase distribution convergence for the GDSS signals, similar to the noise-only case, whereas the DSSS signals generated a phase distribution with a distinct shape, indicating the presence of a valid signal. Additionally, the spectral correlation detector applied to the GDSS signal at an SNR of −12 dB showed no identifiable peaks on the spectrum, providing further evidence of the effectiveness of the GDSS scheme and making it a favourable choice for covert communication applications. A semi-analytical calculation of the bit error rate is also presented for the uncoded system. The investigation results show that the GDSS scheme can generate a noise-like signal with reduced identifiable features, making it a superior solution for covert communication. However, achieving this comes at a cost of approximately 2 dB on the signal-to-noise ratio.

## 1. Introduction

Secure communication schemes are essential for transmitting mission-critical information securely and privately, especially in hostile environments. Traditional methods to achieve secure communication can be broadly categorised into three main groups [1]: cryptographic methods, steganographic methods [2,3,4], and physical layer security techniques [5]. Cryptographic methods use encryption and decryption algorithms to prevent unauthorised access, disclosure, and alteration of data. Steganographic methods are primarily focused on concealing confidential information within non-sensitive objects, such as images, audio, and video files. On the other hand, physical layer security techniques aim to bolster the security of wireless communication systems by leveraging the unique properties of wireless channels. However, none of these methods can conceal the evidence of communication or protect against detection of the transmission. Therefore, an adversary can potentially intercept the transmission and take advantage of any vulnerabilities in the communication protocol to gain access to the secure data or launch electronic attacks against the user [6]. The operational needs of secure communication in contested electromagnetic environments go beyond just protecting the transmitted content; it also requires the concealment of the transmission behaviour [7]. This paper presents a new signalling scheme for covert communication. Covert communication, also known as communication with a low probability of detection (LPD), is centered around hiding any evidence of communication to avoid detection. This is achieved by reducing the received signal-to-noise ratio (SNR) at the eavesdropper [8,9,10].

Research published in the field of LPD communications can be categorised into two main areas: (i) information-theoretic aspects of LPD communications and (ii) designing waveforms for LPD communications. Information-theoretic studies focus on determining the fundamental limits of LPD communication in terms of the amount of information that can be conveyed from a transmitter to a receiver subject to a constraint on adversary’s detection error probability [11,12]. The authors in [13] present a square root law (SRL) that defines the constraints and performance limits of LPD communication for the additive white Gaussian noise (AWGN) channel. The SRL law states that covert and reliable communication can be achieved provided no more than $O\sqrt{n}$ bits are transmitted in *n* channel uses. This gives an information rate of $O(1/\sqrt{n})$, which approaches 0 as *n* goes to infinity. Hence, recent studies have focused on developing LPD schemes to obtain a positive information rate [14].

This paper focuses on designing waveforms for LPD communication. For LPD communications, it is desirable to transmit with minimal PSD to hide the transmitted signal under the receiver’s noise floor [7] and have random like characteristics such as non-repetitive features [15], making the signal indistinguishable from thermal white noise present at any receiver [16,17,18]. These properties are needed, as without them the waveform has the potential to be detected using advanced signal processing techniques such as cyclostationary analysis, higher-order moments analysis, energy detection methods, and time-frequency transforms [19,20,21,22,23].

Existing covert communication schemes use spread-spectrum, chaotic theory, or a combination of both to achieve covert communication. The proposed schemes vary: utilising machine learning [18], using noise envelopes to mask the signal [24], chaotic spreading and modulation [25,26], and using the message itself to spread the signal [27]. In addition to different waveform design approaches, there are also other methods proposed in the literature that exploit the uncertainties in the eavesdropping channel [28], noise power [29], transmit time [30], and interference power from friendly jammers [31] to reduce signal detectability and improve information rate [32]. Other non-conventional LPD techniques include methods based on (1) exploiting the multiplicity of users scattered across the wireless network and the channel variations caused by their mobility [33], (2) directional transmission using multiple antennas [34], (3) opportunistic power control similar to conventional power control with an on–off switch that turns off the transmitter when the channel gain falls below a threshold [35], (4) artificial noise generation to disguise the existence of covert channels [36], and (5) millimeter-wave communications that use feature steerable narrow beams operating in the frequency band of 30–300 GHz [37].

The proposed scheme in this paper uses thermal white noise of the system to obscure signals generated by a DSSS transmitter and produce a spread-spectrum waveform that follows a Gaussian distribution (GDSS). The design of the GDSS scheme ensures that the receiver does not need any information about the sequences used to mask the signal to retrieve the message. To the best of our knowledge, this study represents the first investigation into utilising naturally occurring thermal noise for spread-spectrum communication. We conducted a performance evaluation of the proposed GDSS scheme and compared it to the widely used DSSS technique, which has several weaknesses that make it vulnerable to exploitation by adversaries. These vulnerabilities arise from the use of fixed modulation and repeatable spreading sequences.

The paper is structured as follows. Section 2 describes the standard DSSS system, and Section 3 presents the proposed GDSS waveform scheme. In Section 4, we evaluate the LPD performance of the proposed scheme using higher-order moments, a modulation stripping signal detector, and cyclostationary analysis. Section 5 investigates the error-rate performance of both coded and uncoded systems and compares it with DSSS. Furthermore, a numerical expression for the bit error rate performance of the GDSS system is derived and presented in this section. Finally, we conclude the paper in Section 6 and provide limitations of this research and suggestions for future work.

## 2. DSSS Modulation

**Figure 1:**
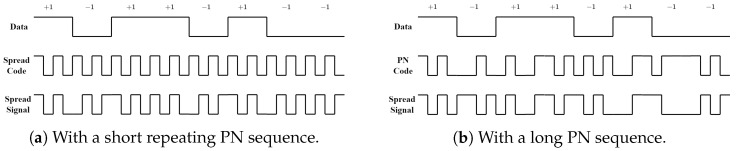
Spreading process of BPSK DSSS with repeating short PN sequence and with a long PN sequence.

DSSS, or direct-sequence spread-spectrum, is a method of modulation in which the message bits are modulated by a pseudorandom bit sequence known as a spreading sequence. This sequence has a much higher rate than the original information rate, and a spreading factor *N* determines the number of spreading bits that map to a message bit. After spreading, the symbols to be transmitted are commonly referred to as chips. When a DSSS system maintains the same bit rate and energy per bit as before spreading, the signal bandwidth will be spread by a factor of *N*, and the magnitude of the PSD of the signal will be reduced by a factor of *N*. This reduction in PSD helps to mitigate interference from other signals. To spread the signal, the data are multiplied by a pseudo-noise (PN) sequence. This PN spreading sequence is unique to each transmitter and receiver pair and helps to ensure secure communication. A binary phase shift keying (BPSK) DSSS spreading process is illustrated in Figure 1.

DSSS is an important technology for covert communications because it enables the system to operate under the thermal noise floor even at low SNR levels (i.e., much lower than 0 dB SNR). This means that the communication signal can be hidden in the noise, making it difficult for adversaries to detect and intercept the signal. The spreading process in DSSS spreads the signal across a wide bandwidth, which reduces the PSD of the signal. As a result, the signal is less susceptible to noise and interference, making it easier to detect at low SNR levels. This is particularly important for covert communications, where it is essential to maintain a low profile and avoid unfriendly detection.

The bit error rate (BER) performance of a DSSS system using quadrature phase shift keying (QPSK) spreading is given by

(1) $$\mathrm{BER}=0.5\;\mathrm{erfc}\left((\sqrt{N(E_{s}/N_{0})/2}\right)$$

where erfc() is the complementary error function and $E_{s}/N_{0}$ is the energy per channel symbol to noise power spectral density ratio. This measure will also be referred to as signal-to-noise ratio (SNR) in this paper.

**Figure 2:**
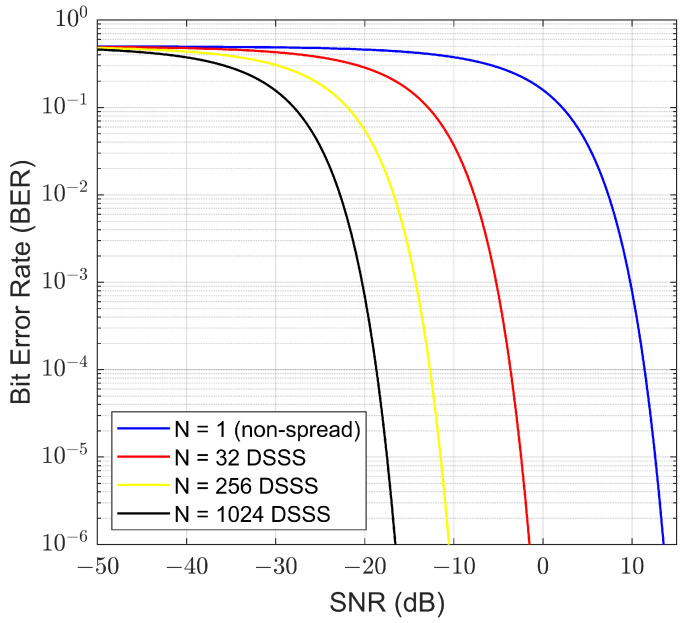
Theoretical BER performance of a DSSS-QPSK system with various spreading factors.

Examples for various spreading factors are shown in Figure 2. Figure 2 shows that the non-spread system achieves a target BER of $10^{-5}$ at 12.5 dB, whereas the DSSS system with a spreading factor of 256 achieves the same BER at −11.5 dB. It is further shown that as the spreading length increases, so does the system’s ability to operate deeper in the noise floor. However, an increase of the spreading factor also causes a reduction in the bit rate if the system bandwidth is maintained fixed.

**Figure 3:**
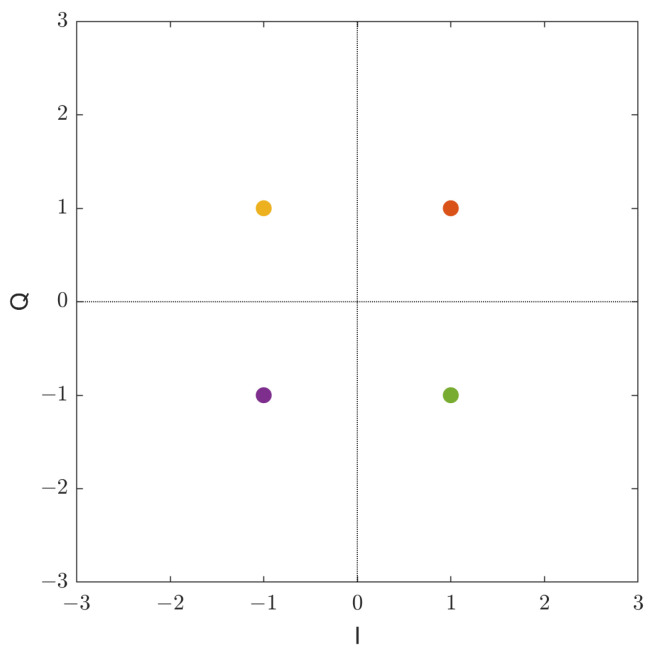
Signal constellation of the DSSS-PN QPSK noise-free transmit signal, colour coded for each position in a quadrant.

The standard DSSS system has several vulnerabilities and may not be ideal for covert communication. This system normally uses repeating patterns and has deterministic features that could be used for detection/interception. For example, Figure 3 shows the constellation of a typical DSSS-PN system with QPSK spreading. The points are color coded for future reference. As the amplitude and phase of the constellation points are fixed, they present deterministic features to the signal, making it easier for an adversary to detect the existence of the signal and possibly intercept the message using advanced signal processing methods [21]. The autocorrelation function (ACF) can be used to measure repeatable patterns of the PN sequences in a DSSS system. Some DSSS systems tackle the issue of predictable PN sequences by using a shared secret key in order to randomly generate the PN sequences [38]. However, implementation of such architectures for covert communication is difficult [39].

## 3. Proposed GDSS Scheme

**Figure 4:**
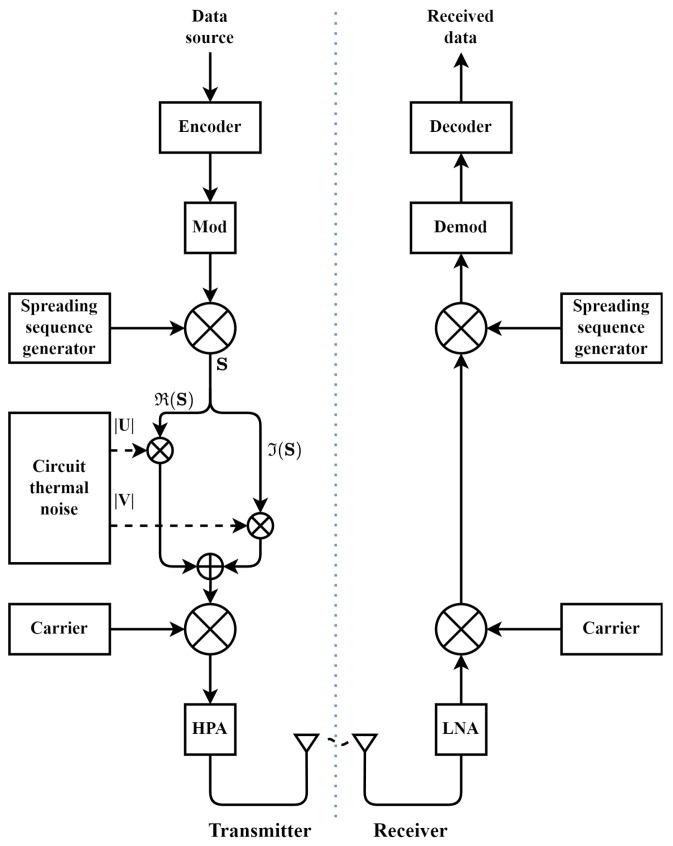
Proposed GDSS system.

The block diagram of the proposed GDSS scheme is illustrated in Figure 4.

The proposed scheme builds upon the standard QPSK DSSS-PN system by utilising the naturally occurring thermal white noise from the transmitter’s circuity. The independently obtained noise sequences are applied to the in-phase (I) and quadrature-phase (Q) components of the signal after spreading to perform the Gaussian masking. The Gaussian masking process for the scheme illustrated in Figure 4 can be expressed as follows. For each QPSK spread complex chip **S**, a complex value U + jV is taken from a transmitter’s circuitry with amplified thermal white noise. Let I and Q be the result of element wise multiplication such that I=ℜ(S)*|U| and Q=ℑ(S)*|V|, where ℜ(S) and ℑ(S) are the real and imaginary components of S, respectively. The resulting product to be transmitted is then expressed as I+jQ. The generated IQ values are upsampled, filtered, modulated with a RF carrier wave, amplified with a high power amplifier (HPA), and transmitted through the antenna. On the receiver side, the received signal is first amplified with a low noise amplifier (LNA) and passed through the RF demodulation and despreading steps to recover the transmitted message.

**Figure 5:**
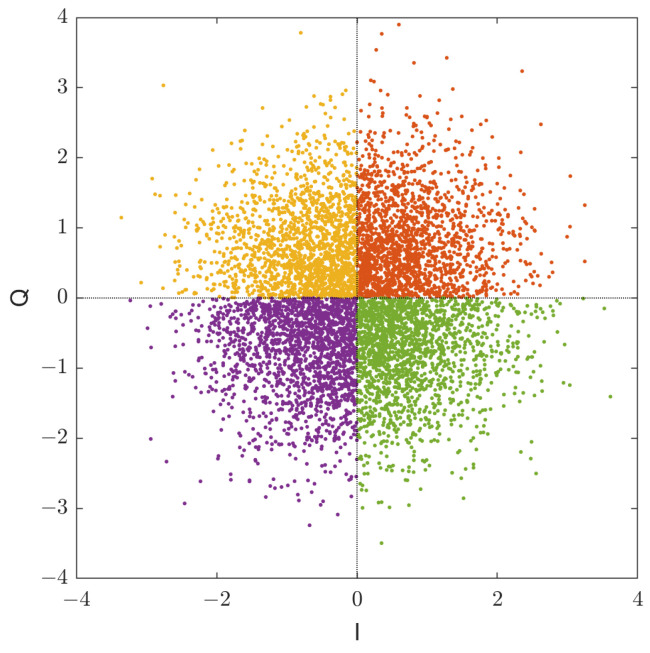
A collection of scattered constellation points of the proposed QPSK GDSS signal, with matching colours of QPSK DSSS in Figure 3.

A snapshot of the varying constellation points generated by masking the fixed QPSK points is shown in Figure 5, where each QPSK chip symbol (in a quadrant) is mapped to a non-deterministic location within the same quadrant every time. The Gaussian masking process moves the original symbol position in Figure 3 by the absolute values of the measured I and Q thermal noise values. It means the noise masking process does not move the symbol into a different quadrant, as illustrated in the colour matching scheme between the two figures. However, the cost of this method is an increase in the system BER, or for a same BER, an increase of the TX power. For a same average TX power, some symbols would be more susceptible to noise than they originally were due to being moved closer to the quadrant boundaries - a decreased Euclidean distance. The impacts to the BER will be quantified later. However, the adversaries detectablility is not necessarily scarified, depending on the detection method.

### Gaussianity of GDSS Signals

**Figure 6:**
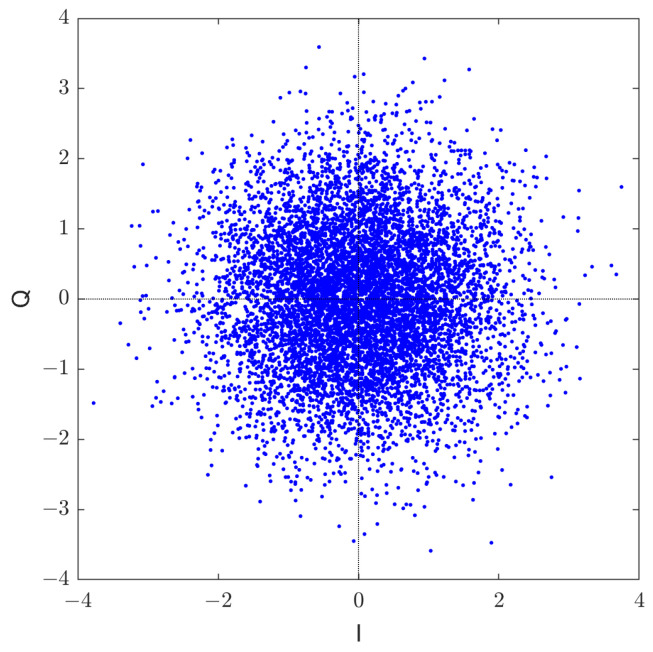
A collection of signal constellations of noise-free transmitted GDSS signal.

This section compares the distribution of the GDSS signals with the theoretical Gaussian distribution. The noise-free signal constellation of the GDSS scheme is shown in Figure 6. The figure shows that the signal constellation is sufficiently masked to appear as chaotic white noise to any adversary.

The probability density function of a random variable *Z* with Gaussian distribution is given by

(2) $$f_{Z}\left(x\right)=\frac{1}{\sqrt{2\pi }}exp^{-x^{2}/2}.$$

Using Equation (2) its moments can be computed using

(3) $$\mu_{k}=\int_{\infty }^{-\infty }x^{k}f_{Z}\left(x\right)dx.$$

A closed-form expression of Equation (3) for even orders of moments of the zero-mean unit-variance normal distribution can be expressed as [40]

(4) $$\mu_{k}=\frac{k!}{2^{k/2}\left(k/2\right)!}$$

**Figure 7:**
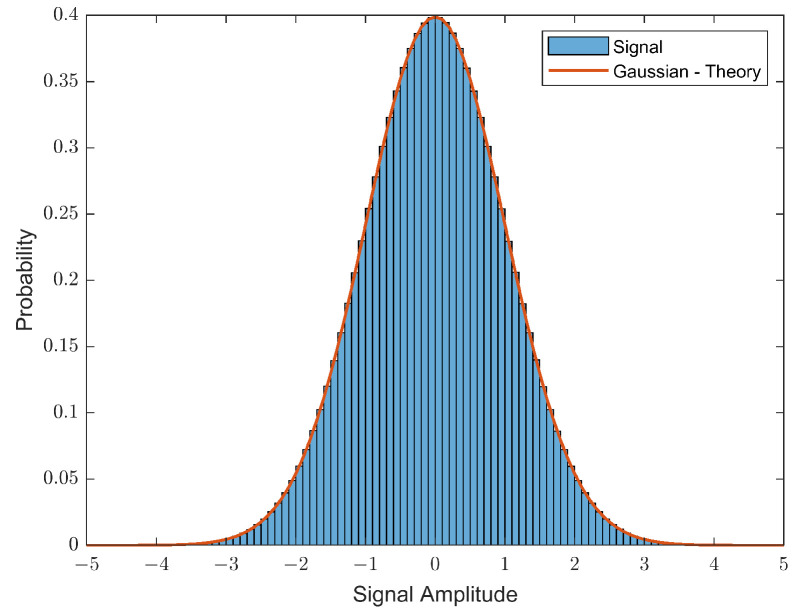
Distribution of the noise-free transmitted GDSS signal.

The transmitted noise free signal is compared against a standard Gaussian distribution (SGD) in Figure 7 and in Table 1 using 1 × $10^{6}$ bits with a spreading factor of 256. The probability distribution shown in Figure 7 closely matches the theoretical Gaussian distribution. The Gaussian distribution is further supported in Table 1 from the estimated moments of the waveform depicting acceptable levels of commonality with the theoretical moments (calculated using Equation (4)).

A random white noise signal would have an ACF of zero at all lags except a value of unity at lag zero, to indicate that the signal is uncorrelated and does not exhibit repetitive time-domain features. The repetitive features of the GDSS signals were analysed using the ACF. The results obtained showed no identifiable features for either GDSS or DSSS noise-free signals provided each uses non-repeating spreading sequences. However, for the case when these systems use a fixed pair of spreading sequences, the ACF for DSSS shows several very strong identifiable components across multiple lags. Compared to this, for the GDSS, the correlation magnitudes are small and are overall at an insignificant level.

## 4. Detectability of GDSS Signals

This section investigates detectability of the GDSS waveform at low SNR using higher-order moments and modulation stripping and compares the performance with DSSS signals. Simulated noise is generated using a random number generator to create a sequence of values that mimic the behaviour of real-world noise. The noise power is adjusted based on the desired SNR value to create noisy signals at different SNR levels.

### 4.1. Detection Using High-Order Moments

**Figure 8:**
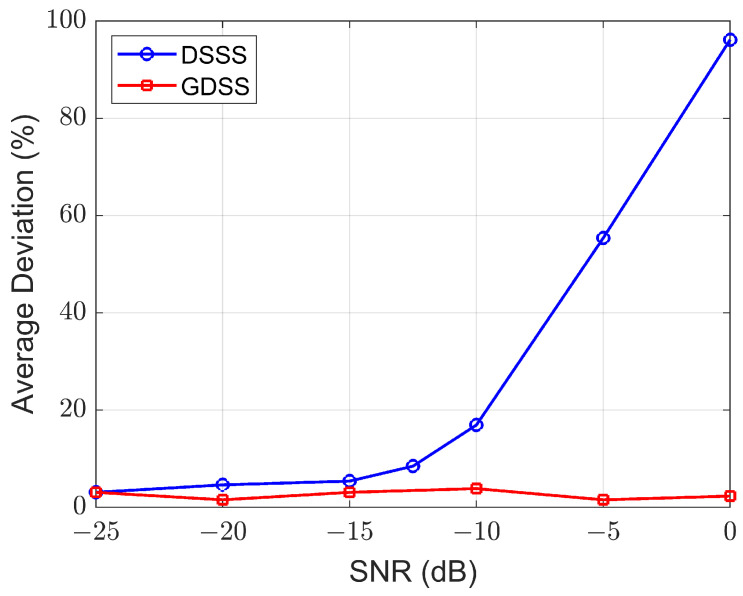
Average deviation of the estimated 20th-order moment from theory for *N* = 256.

Moments are statistical parameters that can be used to measure Gaussian distribution as discussed in the previous section. In this section, the 20th-order moment is used to assess the detectability of the GDSS and DSSS signals operating under the noise floor. The 20th-order moment of the zero-mean unit-variance Gaussian distribution is 654,729,075. The moments of the GDSS and DSSS signal components are estimated at various SNR and then compared with the theoretical moment value using the absolute deviation between the estimated and theoretical values. The results are generated by averaging results from five tests and expressing the average deviation as a percentage of the theoretical value. Each test result is conducted using a spreading factor of 256 on 1 × $10^{6}$ message bits. The signal values are first standardised using Equation (5) to have a mean of 0 and a standard deviation of 1 before estimating the moment:

(5) $$z_{i}=\frac{r_{i}-\bar{r}}{\sigma }$$

where $\bar{r}$ and σ denote the mean and standard deviation of the signal values, respectively. The average deviation measures are plotted in Figure 8.

This figure shows that the average deviation of the DSSS signals are significantly large compared to GDSS values, indicating non-Gaussian features of the DSSS signal even in the presence of high noise levels. Unlike DSSS signals, the measures obtained for the GDSS signals show deviation values very close to zero across all SNR values.

Assuming an average deviation threshold of 10% (to achieve detection with high confidence), the moment-based detector will fail to detect the presence of a GDSS signal, whereas the same detector can easily detect the DSSS signals for SNR > −12 dB. It should be noted that the standard DSSS signal can be Gaussian distributed if the system operates at a sufficiently low SNR. However, this would require an increase in the spreading factor, resulting in a reduction of data rate.

### 4.2. Detection Using Modulation Stripping

**Figure 9:**
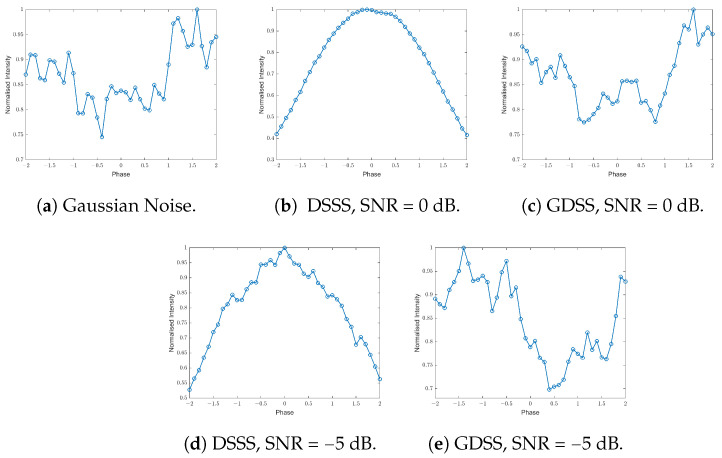
Phase angle modulation stripping detector with QPSK, normalised intensity versus phase, *N* = 256.

In this section, a simple modulation stripping method is used to evaluate the detection performance of the GDSS signals. A non-linearity can be applied to the signal to produce discrete spectral spurs to aid detection. As an example, in the case of QPSK modulation, a fourth power produces a discrete spur at four times the carrier frequency offset. Figure 9 shows the distribution plots obtained for both GDSS and DSSS signals at different SNRs. The distribution plot generated from the detector for a set of Gaussian distributed complex values (Gaussian noise) is presented as a reference in Figure 9a. It should be noted that the developed GDSS scheme in Figure 9c,e also appear random with no significant phase distribution convergence. However, Figure 9b,d show a bell shaped distribution for the DSSS. This means that the phase distribution converges to a particular point, which gives evidence for a set constellation scheme being used, as opposed to the GDSS scheme and Gaussian noise.

### 4.3. Energy Detector

Energy detection is another commonly used method for detecting communication signals. The basic idea behind energy detection is to compare the energy of the received signal to a pre-defined threshold. If the energy of the received signal exceeds this threshold, the signal is assumed to be present. One of the advantages of the energy detection method is its simplicity and low computational complexity. However, energy detection can suffer from high false alarm rates in low SNR environments, where the noise can easily exceed the detection threshold. Therefore, energy detectors are generally not effective for detecting signals, such as DSSS and GDSS signals, operating under the noise floor. As the GDSS system requires more power than the DSSS system to achieve the same BER performance, it is expected that GDSS signals can be more likely detected compared to DSSS signals using an energy detector in high SNR conditions.

### 4.4. Cyclostationary Analysis

Signal detectors based on cyclostationary analysis offer superior detection performance compared to energy detectors in low SNR environments. However, they may require more computational resources and expertise in signal properties. Cyclostationary analysis is a powerful technique used for detecting and analysing communication signals by leveraging their cyclostationary properties. These properties refer to repetitive characteristics of the signal, such as a constant modulation and coding scheme or a fixed synchronisation sequence embedded in the communication waveform. By exploiting these properties, cyclostationary analysis can provide more reliable detection and analysis of signals in challenging environments with low SNR.

Cyclostationary analysis normally involves estimating the spectral correlation function (SCF) of a signal. The SCF is a measure of the correlation between different frequency components of the signal, as a function of frequency offset and time lag. In a cyclostationary signal, the SCF exhibits peaks at certain frequency offsets and time lags, known as cyclic frequencies and cycle periods, respectively. These peaks correspond to the cyclostationary properties of the signal and can be used to detect the presence of the signal operating under the noise floor. By exploiting these peaks, cyclostationary analysis can offer a more robust and reliable detection of weak signals.

**Figure 10:**
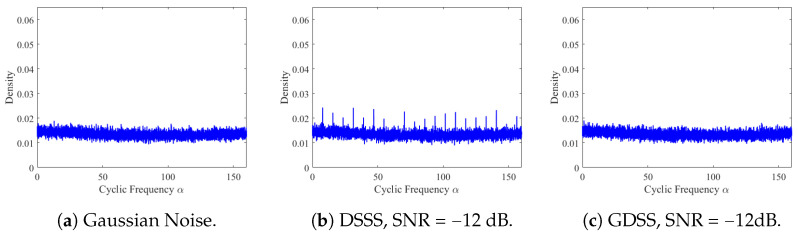
Spectral correlation spectrum for various signals, *N* = 256.

This section aims to validate the effectiveness of GDSS signals in hiding under the noise floor using the fast spectral correlation method proposed in [41]. We present a comparison of the results obtained with the DSSS signal, as shown in Figure 10.

The DSSS signal is generated using a repetitive spreading sequence, and the Gaussian masking process is applied to develop the GDSS signal. To ensure a fair comparison, we keep the observation period and spreading factor constant for both the DSSS and GDSS signals. By analysing the spectral correlation of the two signals, we demonstrate that the GDSS signal provides superior hiding capabilities compared to the DSSS signal under similar conditions. Our findings highlight the potential of GDSS signals for use in low-power and covert communication systems where signal detection and interception are critical concerns.

The spectral correlation density plots shown in Figure 10 illustrate the significant difference between the DSSS and GDSS signals. The DSSS signal exhibits prominent peaks in its spectrum, indicating the presence of repetitive spreading sequences and modulation. In contrast, the application of the Gaussian masking process to the DSSS signal has almost entirely eliminated these peaks. As a result, the GDSS spectrum closely resembles the spectral correlation spectrum generated for the noise-only case (i.e., without any signal). GDSS signal exhibits a much flatter spectrum, with no noticeable peaks. These findings provide strong evidence supporting the superiority of the GDSS method over the DSSS. By eliminating the characteristic peaks of the DSSS signal, the GDSS signal is much harder to detect and intercept, making it a better choice for covert communication systems.

## 5. BER Performance

This section investigates the BER performance of the uncoded and coded GDSS systems and compares them with the corresponding DSSS systems.

### 5.1. Uncoded BER Performance

**Figure 11:**
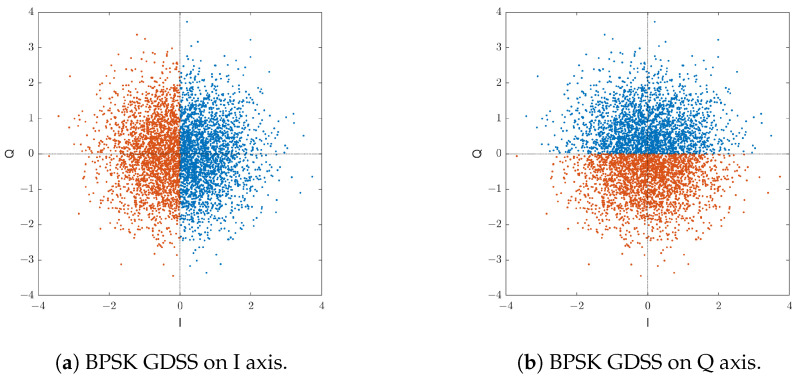
Component BPSK GDSS scatters of the QPSK GDSS signal.

In this section we develop a semi-numerical calculation of the BER performance for the uncoded GDSS system and compare it with the simulated results. The BER performance can be analysed from the I or Q component. Assuming Gray coding is used, the two components are equivalent to two independent BPSK GDSS schemes (Figure 11). The BER performance of the QPSK GDSS is identical to the BPSK GDSS in terms of energy per bit to noise power spectral density ratio ($E_{b}/N_{0}$) or 3dB worse in terms of SNR.

Our objective is to determine the probability density function (PDF) of the decision variable *z*, which is the result of the de-spreading process using a binary spreading sequence (rather than a Gaussian sequence). Here, *z* is the sum of *N* noisy chips during a bit period

(6) $$z=\sum_{i=1}^{N}r_{i}$$

where $r_{i}=s_{i}+n_{i}$ is a received chip, $s_{i}$ and $n_{i}$ are members of the signal sequence $s\;=\;(s_{1},s_{2},\cdots ,s_{N})$ and noise sequence $n=(n_{1},n_{2},\cdots ,n_{N})$, respectively.

The BER is the probability the decision variable becoming negative due to noise disturbance

(7) $$\mathrm{BER}=\int_{-\infty }^{0}p_{z}\left(z\right)dz$$

In order to find $p_{z}\left(z\right)$, we start at one chip’s PDF. The signal chip $s_{i}$ follows a half-normal distribution [42,43]

(8) $$s_{i}∼p_{s_{i}}\left(x\right)=\left\{\begin{array}{cc} \frac{\sqrt{2}}{\sigma_{s}\sqrt{\pi }}\exp\left(\frac{-x^{2}}{2\sigma_{s}^{2}}\right), & x\geq 0,\\ 0, & x<0 \end{array}\right.$$

The corresponding noise $n_{i}$ follows a Gaussian distribution

(9) $$n_{i}∼p_{n_{i}}\left(x\right)=\frac{1}{\sigma_{n}\sqrt{2\pi }}\exp\left(\frac{-x^{2}}{2\sigma_{n}^{2}}\right)$$

Since $s_{i}$ and $n_{i}$ are mutually independent, the PDF of the sum $r_{i}$ is the convolution of PDF’s of the summands

(10) $$r_{i}∼p_{r_{i}}\left(x\right)=p_{s_{i}}\left(x\right)*p_{n_{i}}\left(x\right)$$

where * denotes a convolution. Using Mathematica, we derive the PDF of one noisy chip

(11) $$p_{r_{i}}\left(x\right)=\frac{\exp\left(\frac{-x^{2}}{2(\sigma_{s}^{2}+\sigma_{n}^{2})}\right)\left(1+\mathrm{erf}\left(\frac{\sigma_{s}x}{\sigma_{n}\sqrt{2(\sigma_{s}^{2}+\sigma_{n}^{2})}}\right)\right)}{\sqrt{2\pi (\sigma_{s}^{2}+\sigma_{n}^{2})}}$$

It can be expressed as a function of SNR. For QPSK GDSS, the SNR in dB is

(12) $$\xi =10\log_{10}\left(\frac{\sigma_{s}^{2}}{\sigma_{n}^{2}}\right)$$

For BER calculations we can let $\sigma_{s}^{2}=1$ without loss of generality. We have

(13) $$p_{r_{i}}\left(x\right)=\frac{\exp\left(\frac{-x^{2}}{1+10^{-\xi /10}}\right)\left(1+\mathrm{erf}\left(\frac{x10^{\xi /20}}{\sqrt{1+10^{-\xi /10}}}\right)\right)}{\sqrt{\pi (1+10^{-\xi /10})}}$$

If we can find the characteristic function of $p_{r_{i}}\left(x\right)$ then $p_{z}\left(z\right)$ is the inverse Fourier transform of the *N*th power of the characteristic function. Regrettably, we are unable to obtain an analytical expression for it. An alternative approach is to use self-convolution. As *z* is the sum of *N* independent $r_{i}$, its PDF is the N−1 self-convolutions

(14) $$p_{z}\left(z\right)=\underset{N}{\underset{︸}{p_{r_{i}}\left(x\right)*p_{r_{i}}\left(x\right)*p_{r_{i}}\left(x\right)*\cdots *p_{r_{i}}\left(x\right)}}$$

where * denotes a convolution. Once again, we were unable to derive an analytical solution and had to resort to numerical convolution techniques. To avoid overflow issues when dealing with large values of *N*, we created a normalized function

(15) $$y_{1}\left(x\right)=p_{r_{i}}\left(x\right)\Delta_{x}$$

so that $\sum_{x}y_{1}\left(x\right)=1$, where $\Delta_{x}$ is the increment of the *x* vector. For the sum of *N* received chips, the PDF is proportional to the N−1 self numerical convolutions (also denoted by * )

(16) $$y_{N}\left(x\right)=\underset{N}{\underset{︸}{y_{1}\left(x\right)*y_{1}\left(x\right)*\cdots *y_{1}\left(x\right)}}=\overset{N-1}{\overset{︷}{*}}y_{1}\left(x\right)$$

Performing N−1 numerical convolutions using brute force computation is both expensive and unnecessary for large values of *N*. Instead, a faster algorithm can be implemented, as illustrated below.

We calculate $m=\lfloor \log_{2}N\rfloor$ pairs of convolutions first:

(17) $$\begin{array}{cc} y_{2}\left(x\right) & =y_{1}\left(x\right)*y_{1}\left(x\right)\\ y_{4}\left(x\right) & =y_{2}\left(x\right)*y_{2}\left(x\right)\\ y_{8}\left(x\right) & =y_{4}\left(x\right)*y_{4}\left(x\right)\\ \cdots \\ y_{2^{m}}\left(x\right) & =y_{2^{m/2}}\left(x\right)*y_{2^{m/2}}\left(x\right) \end{array}$$

If *N* is an integer that is a power of two, our objective has been achieved. However, if *N* lies in the range $2^{m}<N<2^{m+1}$, further convolutions are necessary, using the *m* results from Equation (17) as the foundational building blocks. Only those terms corresponding to the ones of *N* in binary form $N_{bin}$ are required. For example, $N=50=2^{5}+2^{4}+0+0+2^{1}+0$, in binary form it is $N_{bin}=110,010$. Only two more convolutions are required.

(18) $$y_{50}\left(x\right)=y_{2^{5}}\left(x\right)*y_{2^{4}}\left(x\right)*y_{2^{1}}\left(x\right)=y_{32}\left(x\right)*y_{16}\left(x\right)*y_{2}\left(x\right)$$

**Figure 12:**
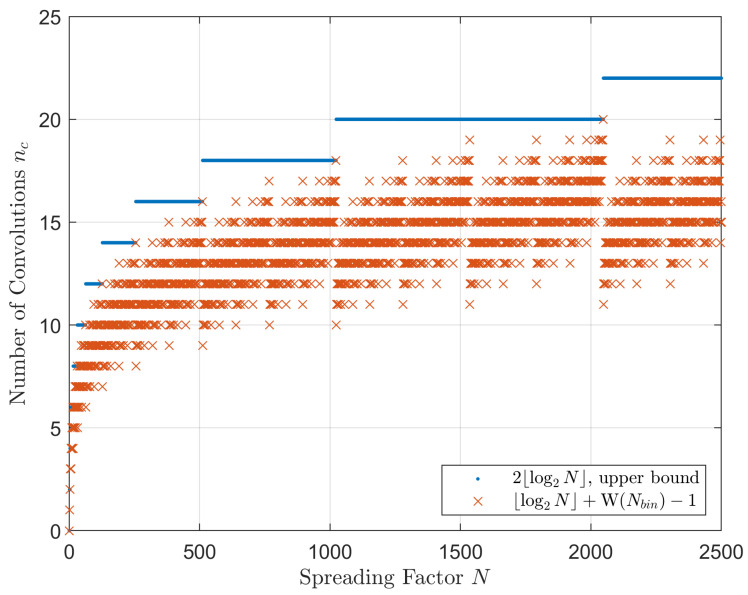
Uncoded GDSS BER computation: number of numerical convolutions $n_{c}$ and its upper bound, versus spreading factor *N*.

We see that instead of 49 convolutions, we only need to conduct 7 of them. In general, the required number of convolutions is

(19) $$n_{c}=\left\lfloor \log_{2}N\right\rfloor +W\left(N_{bin}\right)-1$$

where $W(N_{bin})$ is the binary weight function, producing the sum of ones in the binary number $N_{bin}$. The $n_{c}$ is upper bounded by 2m. These are plotted in Figure 12.

Finally, the PDF for the decision variable, the sum of *N* de-spread chips, is

(20) $$p_{z}\left(z\right)=\frac{y_{N}\left(x\right)}{\Delta_{x}}=\frac{\overset{N-1}{\overset{︷}{*}}y_{1}\left(x\right)}{\Delta_{x}}$$

**Figure 13:**
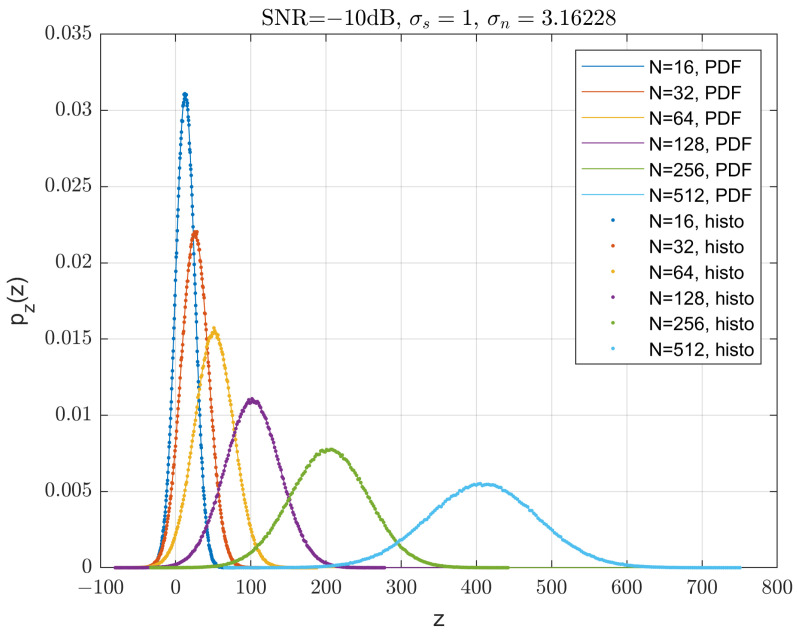
A selection calculated PDFs of the decision variable $p_{z}\left(z\right)$ and Monte Carlo histograms.

Note here we did not distinguish the variables *z* and *x*. Some computed $p_{z}\left(z\right)$ and histograms are plotted in Figure 13. Calculations and simulations agree very well.

In numerical calculations

(21) $$\mathrm{BER}=\sum_{x_{i}=x_{min}}^{0}p_{z}\left(x_{i}\right)\Delta x$$

where $x_{min}$ and Δx need to be chosen carefully to ensure the calculation is valid. To check it, we test if the total sum is one or near one. Practically, we use Δx=0.01 and satisfy

(22) $$\sum_{x_{i}=x_{min}}^{x_{max}}p_{z}\left(x_{i}\right)\Delta x\geq 0.995$$

**Figure 14:**
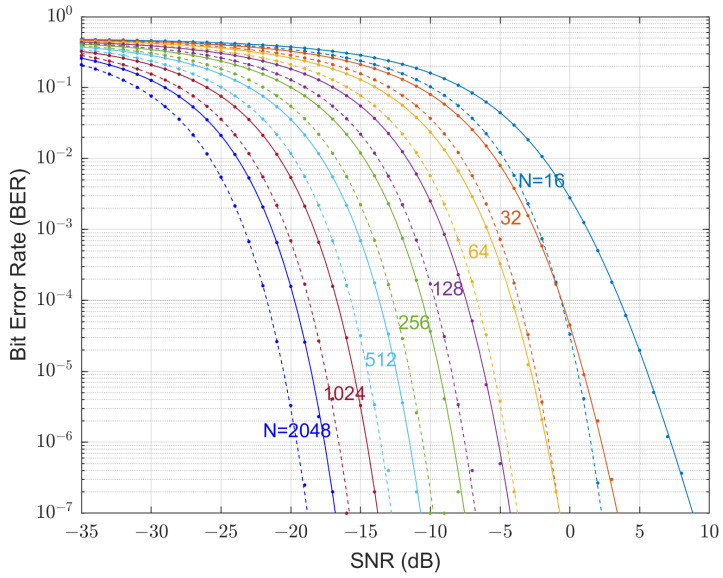
Uncoded BER performance: theoretical QPSK GDSS (solid lines) and QPSK DSSS (dashed lines) with various spreading factor *N*, and corresponding simulation results (dots).

It is only difficult to satisfy this condition at very low SNRs, for example, ξ=−35 dB. For medium low to high SNRs, most sums are equal to 1. It also depends on the spreading factor *N*. The $x_{max}=-x_{min}$ are pre-calculated by a trial-and-error method. For N=[16, 32, 64, 128, 256, 512, 1028, 2048], the $x_{max}$ obtained are $x_{max}=[$7700, 9900, 14300, 10450, 9900, 8800, 5500, 5500]. The batch computation for the 8 BER curves in Figure 14 took about 12 h on a desktop PC with an i7 CPU at 3 GHz.

The BER performance of the uncoded QPSK DSSS systems are also plotted in Figure 14. The theoretic calculations and Monte Carlo simulations agree very well.

The figure shows that the Gaussian distributed scheme performs 2.5 dB worse than DSSS for a spreading factor of N=64 at a target BER of $10^{-5}$. This loss reduces to 2 dB when N≥512.

### 5.2. LDPC-Coded GDSS System

**Figure 15:**
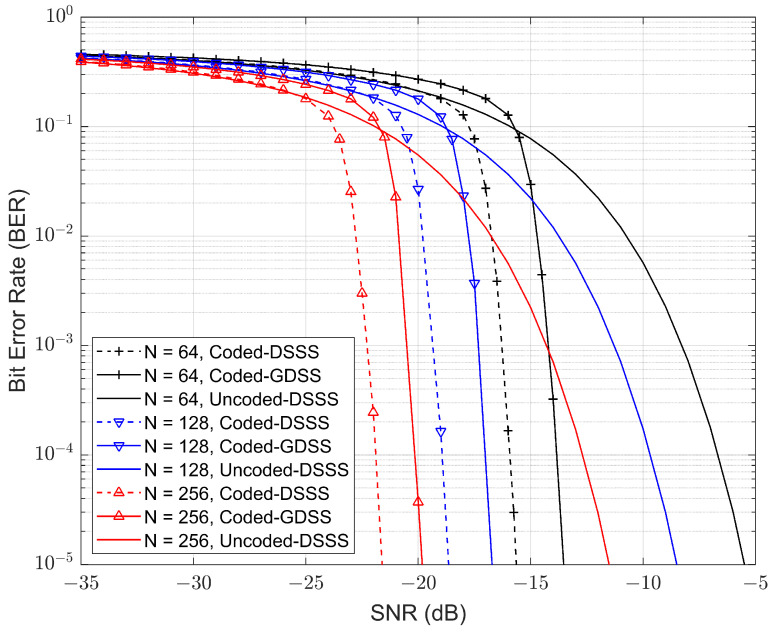
Comparison of LDPC encoded GDSS QPSK system with varying spreading factors against the theoretical DSSS QPSK system’s upper performance bound.

This section investigates the use of low density parity check (LDPC) coding to improve the performance for the GDSS scheme. LDPC codes are a type of linear block error correction codes with parity-check matrices (H) that contain a very small number of non-zero entries. The sparseness of H guarantees the minimum Hamming distance and decoding complexity to increase linearly with the code length. Some of the published results show that these codes can perform close to 0.0045 dB away from the Shannon Limit, making it one of the most powerful error-correction codes known today [44]. To reduce the decoding latency a short half-rate LDPC code with number of message bits k = 324 and codeword length n = 648 bits were used for both the GDSS and the traditional DSSS system. The results obtained are presented in Figure 15.

Figure 15 compares SNR for fixed bandwidth, i.e., the same chip rate in each case. The 1/2-rate coded-GDSS [N = 128] scheme has a power advantage of approximately 5 dB against the uncoded DSSS [N = 256] on the basis of identical information bit rate. However, a comparison of both coded systems (coded-GDSS with coded-DSSS) showed similar performance reduction that was observed for the uncoded system comparison in Section 5.1.

## 6. Conclusions

In this paper, we propose a novel signalling scheme called GDSS for covert (LPD) communications, which aims to conceal the existence of wireless transmissions for enhanced security compared to cryptography and steganography-based schemes. We evaluate the performance of GDSS and compare it with the commonly used DSSS technique, which, despite its popularity, is susceptible to exploitation by adversaries due to various weaknesses. These vulnerabilities stem from the use of fixed modulation and repeatable spreading sequences. In contrast, the proposed GDSS scheme takes advantage of naturally occurring thermal noise at the transmitter to create non-repetitive, featureless signals for communication, which eliminates many of the shortcomings associated with DSSS. In this paper, the proposed GDSS scheme is compared with the DSSS system. The findings indicated that the signals produced by the GDSS approach have fewer distinctive characteristics compared to the DSSS signals, resulting in a reduced likelihood of detection for anyone attempting to intercept the transmission. The Gaussianity test demonstrated that the distribution of the noise-free GDSS signals closely resembled that of the naturally occurring noise in the receiver. To assess the detectability of the signals generated by the proposed scheme by an adversary, this study utilised three detectors: a high-order moments based detector, a modulation stripping detector, and a detector based on cyclostationary analysis. These detectors are applied to signals corrupted with noise. The results showed that the moment-based detector failed to detect the GDSS signal with *N* = 256 at all SNRs, whereas it could easily detect the DSSS signals as low as −12 dB. We applied the modulation stripping detector to both the GDSS and DSSS signals at an SNR of 0 dB and −5 dB, respectively. Our analysis revealed that the GDSS signals exhibited no significant phase distribution convergence, similar to the noise-only case (i.e., without any signal). In contrast, the DSSS signals generated a phase distribution with a distinct shape, indicating the presence of a valid signal. Moreover, we applied the spectral correlation detector to the GDSS signal at an SNR of −12 dB. Our analysis showed no identifiable peaks on the spectral correlation spectrum, providing additional evidence of the effectiveness of the GDSS scheme and the lack of distinguishable peaks in the spectrum suggests that the GDSS signal is difficult to detect and intercept, making it an ideal choice for covert communication applications.

A quasi-analytical expression is also used to derive the BER for the uncoded GDSS, and the results were consistent with the simulation. The comparison of BER between the uncoded GDSS and DSSS systems showed a penalty of 2 to 3 dB for GDSS. However, LDPC coding helped recover this loss and significantly improved the overall BER performance. As expected, the LDPC-coded GDSS performed about 2 dB worse than the corresponding LDPC-coded DSSS system.

This paper did not investigate the effects of high power amplifiers on the generated GDSS signals. The GDSS scheme is capable of generating signals with a high peak-to-average power ratio (PAPR), which is a measure of the dynamic range of a signal. Consequently, amplifying GDSS signals can cause distortion or clipping, as the amplifier may not be able to handle the high instantaneous power levels (albeit rarely happen), which can result in reduced signal quality and performance degradation.

In future work, we plan to analyse the information-theoretic optimality of the proposed GDSS scheme and its variants. We would also like to investigate the impact of real-world factors such as radio impairments, channel effects, and receiver synchronisation on the performance of the GDSS system. These factors can significantly affect the quality and reliability of the communication channel, and thus it is crucial to study their effects on the proposed scheme. Additionally, we plan to explore ways to mitigate the high PAPR issue of the GDSS signals, which can lead to performance degradation.

## Figures and Tables

**Table 1:** Moments of GDSS noise-free signal for *N* = 256.

Moment	SGD (Theory)	GDSS	DSSS
2nd	1	1	1
4th	3	3	1
6th	15	15	1
8th	105	105	1
10th	945	947	1
12th	10,395	10,428	1
14th	135,135	135,524	1
16th	2,027,025	2,025,880	1
18th	34,459,425	34,102,797	1
20th	654,729,075	634,790,833	1

## Data Availability

Not applicable.
